# Technology innovation and environmental outcomes of road transportation policy instruments

**DOI:** 10.1038/s41467-025-59111-8

**Published:** 2025-05-14

**Authors:** Clara Ma, Cristina Peñasco, Laura Díaz Anadón

**Affiliations:** 1 Centre for Environment, Energy and Natural Resource Governance, Department of Land Economy, University of Cambridge, Cambridge, UK; 2 Conservation Research Institute, University of Cambridge, Cambridge, UK; 3 Department of Politics and International Studies, University of Cambridge, Cambridge, UK; 4 Centre for Climate and Nature, Banque de France, Paris, France; 5 Belfer Center for Science and International Affairs, Harvard Kennedy School, Harvard University, Cambridge, MA USA

**Keywords:** Climate-change policy, Sustainability, Environmental impact

## Abstract

Road transportation policies can drive innovation in more environmentally sustainable vehicle and fuel technologies but may have unintended consequences. To assess their impacts on technology innovation, greenhouse gas emissions, air pollution, and land use, we systematically review and analyze evidence on the outcomes of 14 road transportation policy instruments, including fuel economy and low-carbon fuel standards, biofuel and zero-emission vehicle mandates, and fuel and vehicle taxes. We find that the effects of these policy instruments depend on their interactions, design, choice, and sequencing. We identify six types of relationships between policy instruments and highlight design features that have inadvertently increased vehicle emissions. We trace the evolution of electric vehicles through policy milestones shaped by experimentation and competition among influential jurisdictions based on their domestic priorities, industrial structure, and incumbent industry resistance. We show that policy instruments promoting first-generation biofuels have in some cases inhibited innovation in advanced biofuels.

## Abstract (web summary)

Effective road transportation policies are key to tackling climate change and air pollution. This study assesses how the design, interactions, choice, and sequencing of 14 policy instruments affect innovation and environmental policy outcomes.

## Introduction

Conventional transportation systems are among the largest contributors to greenhouse gas emissions and air pollution globally, with road vehicles responsible for a majority of this impact^1^. Exposure to air pollution from road transportation is strongly linked to increased mortality^2^, and exhaust emissions alone are estimated to cause hundreds of thousands of deaths each year^3^. Effective policy interventions can support a sustainability transition^4,5^ in road transportation by accelerating the diffusion of cleaner technologies and encouraging shifts towards public and active modes of transport, helping to reduce the sector’s environmental impact and public health burden as well as meet climate goals^6^.

The automotive industry—a strategically important sector in many countries—relies substantially on government intervention to foster demand for more sustainable technologies, overcome long development cycles, challenge industry incumbents, and gain consumer acceptance^7^. Governments have used policy instruments (e.g., regulatory standards, mandates, economic incentives, and taxes) to encourage automakers, manufacturers, and suppliers to develop more efficient vehicle and fuel technologies, and consumers to adopt them. However, policy instruments may have undesired consequences where technology innovation and environmental objectives are misaligned or result in trade-offs that undermine progress towards one or more of these objectives^8^.

In this systematic review, we analyze the technology innovation and environmental outcomes of policy instruments in the road transportation sector (Table 1), with an emphasis on the impact that these policy instruments have on the development and deployment of vehicle and alternative fuel technologies. Our work responds to calls to investigate the link between different types of policy instruments and technology innovation in the context of transportation^9^, considering policy instrument interactions and design features that are responsible for or could help avoid negative environmental outcomes and trade-offs^6^. Previous research in this area has not systematically accounted for interactions between road transportation policy instruments and has largely focused on one type of policy instrument, on singular measures of innovation (e.g., patents) without considering environmental impacts or trade-offs between outcomes, or not on the transportation sector^7,10,11^. Our review addresses the gap in understanding in terms of how various policy instruments influence technology innovation and environmental sustainability in road transportation, focusing on interactions between different types of policy instruments, design features of policy instruments, and the selection and sequencing of policy instruments.

**Table 1:** Taxonomy of policy instruments included in systematic review

Regulatory Instruments	Performance Standards	Fuel Economy Standard (FES)
		Vehicle Emissions Standard (VES)
		Low-Carbon Fuel Standard (LCFS)
	Technology Standards	ZEV Mandate (ZEV)
		Biofuel Mandate (BM)
Economic Instruments	Taxes	Vehicle Tax (VT)
		Road Tax (RT)
		Fuel Tax (FT)
	Subsidies	Vehicle Subsidy (VS)
		Scrappage Incentive (SI)
		Biofuel Subsidy (BS)
	Technology Investment	R&D Funding (RDF)
		Public Procurement (PP)
	Infrastructure Investment	Charging Infrastructure (CI)

See Supplementary Table 1 for definitions. Source: own elaboration.

Drawing from the energy technology innovation systems and environmental and transport policy evaluation literatures^12–14^, we develop an indicator-based framework to systematically review, analyze, and synthesize evidence from 468 peer-reviewed studies on the technology innovation and environmental outcomes of 14 types of regulatory and economic policy instruments related to road transportation (Table 1). Within these categories of policy instruments, we make further distinctions between performance standards, technology standards, taxes, subsidies, and technology and infrastructure investment (see Supplementary Table 1 for policy instrument definitions).

The technology innovation outcomes reviewed (Table 2) span the innovation process from early-stage research, development, and demonstration to deployment and diffusion (see Supplementary Table 2 for outcome definitions). The indicators for these outcomes include direct outputs of the innovation process, like patents and publications, as well as indicators representing downstream economic consequences of policy instruments, like vehicle sales and biofuel production volumes, which are associated with later stages of innovation. Environmental outcomes include air pollution, greenhouse gas emissions, and land use. In the rest of this paper, “outcome” refers to the set of technology innovation and environmental outcomes in Table 2 and “impact” refers to the direction of a policy instrument’s effect on one of these outcomes (positive, negative, or null).

**Table 2:** Typology of indicators for  policy instrument evaluation

Innovation Inputs	R&D Spending (RD)
Innovation Outputs	Patents, Publications, and Prototypes (PA)
Innovation Outcomes	Modular Improvements (MI)
	Fuel Economy (FE)
	Vehicle Sales (SA)
	Biofuel Production (BP)
	Technology Transfer (TT)
	Cost Reductions (CR)
Environmental Outcomes	Greenhouse Gas Emissions (GG)
	Air Pollution (AP)
	Land Use (LU)

See Supplementary Table 2 for definitions. Source: own elaboration.

In order to characterize the effects of the different policy instruments on innovation outcomes, we group the former according to their impacts on conventional or advanced vehicle and biofuel technologies (see Table 3 for a categorization of these technologies). We use “conventional” to denote internal combustion engine vehicles (i.e., gasoline- and diesel-powered vehicles) and first-generation biofuels (e.g., corn and sugarcane ethanol), while “advanced” refers to electric vehicles (e.g., battery, plug-in hybrid, hydrogen fuel cell) and second- and third-generation biofuels (e.g., cellulosic and algae biofuels).

**Table 3:** Categorization of vehicle and biofuel technologies

	Conventional	Advanced
Vehicle	Internal combustion engine vehicle technologies (e.g., fuel-efficient gasoline or diesel engine designs, advanced/lightweight materials, tailpipe emissions controls)	Electric vehicles technologies (e.g., battery, hybrid, fuel cell)
Biofuel	First-generation biofuel technologies (e.g., corn/sugarcane ethanol, soy/rapeseed/palm oil biodiesel)	Second- and third-generation biofuels (e.g., lignocellulosic ethanol, algae-based biofuel)

Source: own elaboration.

We assess the scientific literature on the influence of public policy instruments on technology innovation processes in vehicles and alternative fuels (see “Methods” for inclusion and exclusion criteria). We review evidence from a variety of disciplines and include both technology innovation and environmental outcomes of road transportation policy instruments, which enables a closer examination of areas where there are synergies and trade-offs between technology innovation and sustainability goals as well as between outcomes representing different stages of the innovation process, different types of technologies, and different environmental outcomes (i.e., greenhouse gas emissions, air pollution, and land use).

Drawing from the evidence we have reviewed, we show how policy instrument interactions, design, choice, and sequencing affect technology innovation and environmental outcomes in road transportation. We discuss ways for policymakers to exploit synergies and mitigate observed and potential trade-offs between policy instruments and their associated outcomes—through policy instrument design, selection, and sequencing—to mobilize technology innovation towards improving environmental sustainability in road transportation.

## Results

**Fig. 1:**
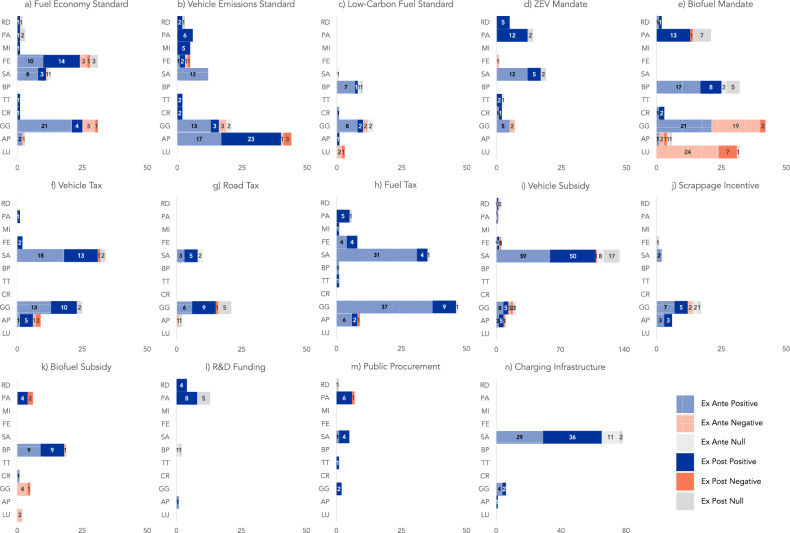
Overview of road transportation policy instrument impacts on technology innovation and environmental indicators. The figure shows the impacts of 14 policy instruments (**a**–**n**; see Supplementary Table 1 for policy instrument definitions) on 11 outcomes (on the vertical axis) based on the direction of the impact (positive, negative, or null) and evaluation type (ex ante or ex post). The number of evaluations associated with each policy instrument and outcome are shown on the horizontal axis. Outcomes on the vertical axis for each plot, from top to bottom, are research and development spending (RD); patents, publications, and prototypes (PA); modular improvements (MI); fuel economy (FE); vehicle sales (SA); biofuel production (BP); technology transfer (TT); cost reductions (CR); greenhouse gas emissions (GG); air pollution (AP) and land use (LU) (see Supplementary Table 2 for outcome definitions). Positive-impact, negative-impact, and null-impact evaluations are in blue, orange, and gray, respectively. Ex ante evaluations are denoted by the dotted pattern and ex post evaluations by the solid-colored bars. The figure summarizes the impacts of regulatory policy instruments (**a**–**e**), economic instruments (**f**–**n**), which we have coded from the reviewed evidence. Note that horizontal axes use the same scale for all figures except for **i** vehicle subsidy and **n** charging infrastructure. Supplementary Data 3 presents the information in this figure in table form.

The final literature sample includes 468 peer-reviewed ex ante and ex post studies comprising more than 1000 evaluations of policy instruments (see “Methods” and SI.1 for a detailed elaboration of the systematic review process). Ex ante studies use quantitative methods, including theoretical model simulations, stated preference surveys, and consequential life cycle assessments. Ex post studies use both quasi-experimental and qualitative methods, including econometric panel data analysis, case studies, and revealed preference surveys. Evidence identified during the systematic review was most concentrated in the United States, China, and in European countries. Figure 1 provides an overview of the policy instruments, outcomes, number of evaluations, and the types of impacts that we extracted and coded from the literature. There are marked differences between the outcomes that have been studied for different policy instruments, and there are numerous combinations of policy instruments and outcomes with little to no evidence. For example, many policy instruments have no evaluations for the technology transfer indicator, and some policy instruments, such as the low-carbon fuel standard (Fig. 1c) and road tax (Fig. 1g), do not have evaluations for patents. Figure 1 also shows that there is little research that attempts to isolate the relationship between policy instruments and cost reductions, an important indicator of learning processes associated with technology diffusion (CR in Fig. 1). The figure highlights several areas in which there are conflicting results in terms of the direction of the impact of policy instruments, for example for vehicle taxes and air pollution, where there are both positive- and negative-impact evaluations (Fig. 1f), and for biofuel mandates and greenhouse gas emissions, where ex ante evidence is mixed while ex post evidence is negative (Fig. 1e). In general, ex post evidence suggests that policy instruments expanding first-generation biofuel production have increased greenhouse gas emissions and land use (Fig. 1e, k). With some exceptions, ex ante and ex post evaluations are broadly consistent in terms of the direction of the impact across most policy instruments and outcomes (Fig. 1). We explore conflicts in policy instrument impacts in the following sections.

### Policy instrument interactions

Understanding interactions between individual policy instruments is key to building more effective policy mixes for sustainability transitions in general^9,15^ and for road transportation in particular^6,16^. Combining two or more instruments can amplify, diminish, or have no impact on their combined effectiveness in terms of promoting technology innovation or environmental sustainability as compared with their effects in isolation^17^. Based on the evidence in our sample, we present six types of positive, negative, and neutral relationships between road transportation policy instruments: (1) pre-condition, (2) synergy, (3) addition, (4) overlap, (5) trade-off, and (6) backfire (Tables 4 and 5). The policy instrument interactions we have extracted from the literature are linked to policy instruments in specific jurisdictions or scenarios and thus are not necessarily generalizable. Although we focus on pairs of different types of policy instruments, interactions can also occur between policy instruments at different administrative levels or in combinations of more than two instruments.

**Table 4:** Road transportation policy instrument interactions and their effects on technology innovation outcomes

	BM	CI	FES	FT	PP	RDS	SI	VS	ZEV
Biofuel Mandate (BM)					$\circleddash$	$\circleddash$			
Charging Infrastructure (CI)					$\oplus$			$\circledast \oplus \oplus$	
Fuel Economy Standard (FES)				$\oplus$				$\circledcirc \,\otimes$	$\otimes \,\otimes$
Fuel Tax (FT)									
Public Procurement (PP)								$\oplus$	
R&D Subsidy (RDS)									
Scrappage Incentive (SI)								$\oplus$	
Vehicle Subsidy (VS)									$\circledcirc$
ZEV Mandate (ZEV)									

This table summarizes interactions between policy instruments and their effects on technology innovation outcomes, as extracted from ex ante and ex post evaluations in the systematic review sample. The circled asterisk $\circledast$, circled plus $\oplus$, circled slash $\oslash$, circled ring $\circledcirc$, circled dash $\circleddash$, and circled times $\otimes$ symbols represent pre-condition, synergy, addition, overlap, trade-off, and backfire policy instrument interactions, respectively, ordered from most positive to most negative. The number of symbols reflects the number of evaluations associated with each interaction effect. See Supplementary Data 1 for additional information on policy instrument interactions.

$\circledast$ Pre-Condition $\oplus$ Synergy $\oslash$ Addition $\circledcirc$ Overlap $\circleddash$ Trade-Off $\otimes \,$Backfire.

**Table 5:** Road transportation policy instrument interactions and their effects on environmental outcomes

	BM	BS	FES	FT	LCFS	RT	SI	VES	VS	VT	ZEV
Biofuel Mandate (BM)		$\otimes \,\,\otimes$			$\circledcirc$						
Biofuel Subsidy (BS)											
Fuel Economy Standard (FES)				$\oplus \oplus \circledcirc$		$\circledcirc$			$\circledcirc \,\, \otimes$	$\circledcirc$	$\oplus \otimes \otimes$
Fuel Tax (FT)						$\circledcirc$		$\circleddash$	$\circledcirc$	$\circledcirc$	$\oplus$
Low-Carbon Fuel Standard (LCFS)											$\circledcirc\, \oslash$
Road Tax (RT)									$\circledcirc$	$\circledcirc$	$\oplus$
Scrappage Incentive (SI)								$\oplus$			
Vehicle Emissions Standard (VES)										$\circledcirc \, \circledcirc$	
Vehicle Subsidy (VS)										$\circledcirc$	$\oplus$
Vehicle Tax (VT)											$\oplus$
ZEV Mandate (ZEV)											

This table summarizes interactions between policy instruments and their effects on environmental outcomes, as extracted from ex ante and ex post evaluations in the systematic review sample. The circled asterisk $\circledast$, circled plus $\oplus$, circled slash $\oslash$, circled ring $\circledcirc$, circled dash $\circleddash$, and circled times $\otimes$ symbols represent pre-condition, synergy, addition, overlap, trade-off, and backfire policy instrument interactions, respectively, ordered from most positive to most negative. The number of symbols reflects the number of evaluations associated with each interaction effect. See Supplementary Data 1 for additional information on policy instrument interactions.

$\circledast$ Pre-Condition $\oplus$ Synergy $\oslash$ Addition $\circledcirc$ Overlap $\circleddash$ Trade-Off $\otimes \,$Backfire.

A positive policy instrument interaction occurs when one policy instrument reinforces another instrument such that the overall impact of the two instruments together is greater than the sum of the impacts of the individual instruments, or when one policy instrument acts as a pre-condition to another instrument. Several studies highlight positive interactions between support for charging infrastructure and subsidies for EVs^18–20^ (Table 4).

A neutral relationship between policy instrument exists when there is no clear interaction between the two policy instruments such that their overall impact is roughly the sum of their individual impacts. We describe these instruments as additive, meaning that they are generally independent and auxiliary to each other. For example, unlike in the light-duty passenger vehicle market where electrification has emerged as a dominant solution, zero-emission vehicle mandates and low-carbon fuel standards could be additive in the road freight sector^21^ in the sense that they do not facilitate, reinforce, or clash with each other, so long as battery technology remains less viable for heavy-duty applications (Table 5).

A negative interaction may occur when the aim or effect of one policy instrument coincides or conflicts with that of another, resulting in overlap (i.e., when the combined effect of two instruments is smaller than the sum of their effects in isolation), trade-off (i.e., when a better outcome in one area is associated with a worse outcome in another), or backfire (i.e., when the combined effect of two instruments is worse than that of the best instrument alone)^17^. State-level low-carbon fuel standards and national-level biofuel mandates in the US, which have intersecting goals, are one example of overlapping policy instruments^22^ (Table 5). The effect of two overlapping instruments combined, however, may still be greater than that of each instrument alone. Because they favor first-generation biofuels, biofuel mandates undercut government R&D subsidies for advanced biofuel technologies, leading to a trade-off^23^ (Table 4). Biofuel mandates may even backfire when combined with biofuel subsidies if gasoline prices are made lower than under the mandate alone, increasing fuel consumption and greenhouse gas emissions^24,25^ (Table 5).

There are also examples of negative policy instrument interactions influencing vehicle technologies. For instance, policy instruments that increase the adoption of EVs may weaken fuel economy and vehicle emissions standards applied to conventional vehicles, particularly when these instruments grant inflated compliance credit to EVs, allowing car manufacturers to offer less-efficient conventional vehicles as a result^26–32^ (Tables 4 and 5).

That a particular combination of policy instruments exhibits negative interactions does not imply that their combined effects are not still greater than the effect of the individual instrument. In a policy mix, a moderately negative combination of instruments may have other justifications for implementation—for instance, because it counteracts rebound effects, enhances political acceptability and cost-effectiveness, or stimulates innovation^16,33^.

### Policy instrument design

**Fig. 2:**
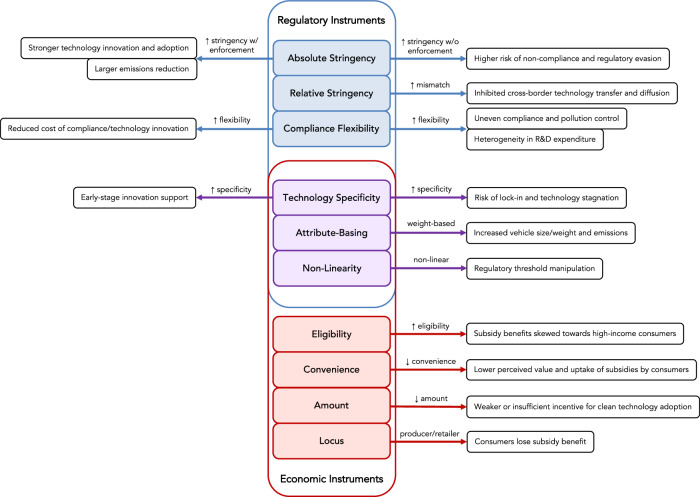
Road transportation policy instrument design features and their effects on technology innovation and environmental outcomes. Regulatory instrument design features are displayed in blue boxes, economic instrument design features in red, and design features applying to both categories of instruments in purple. Positive and negative outcomes associated with these design features, coded from the literature, are shown on the left and right of the figure, respectively. The text above the horizontal arrows describes the mode or direction of the design feature. Vertical arrows pointing upwards and downwards denote increases and decreases, respectively.

We have identified and classified 10 policy instrument design features (Fig. 2) associated with regulatory and economic road transportation policy instruments (Table 1) and below describe how the use of these design features has had both positive and negative effects on technology innovation and adoption, on compliance with emissions and efficiency standards, and on the overall effectiveness of policy instruments in terms of their technology innovation and environmental objectives. For the definition and discussion of the four design features associated with economic policy instruments, see SI.2.

Higher absolute regulatory stringency—the level or limit value of a regulatory standard^34^—often produces stronger technology innovation and adoption outcomes with greater emissions reductions in the jurisdiction in which the standard is implemented^35–39^. An important caveat is that the tightening of standards has resulted in increased non-compliance and cheating by regulated firms in the absence of strong monitoring and enforcement mechanisms^40–43^. For instance, NO_x_ emissions increased after the tightening of the EU vehicle emissions standard due to higher rates of non-compliance by automobile manufacturers and increased emissions from non-compliant vehicles^43^. However, after real-world emissions testing procedures were introduced in South Korea for heavy-duty vehicles between the implementation of Euro 5 and Euro 6 standards, NO_x_ emissions from vehicles subject to the Euro 6 emissions standard decreased substantially as automobile manufacturers developed improvements to exhaust aftertreatment systems^44^, suggesting that improvements in testing conditions can mitigate non-compliance issues when absolute regulatory stringency is increased.

Relative regulatory stringency refers to differences in the stringency or the demands of one set of requirements compared to another in regulatory contexts. For vehicle emissions standards, the difference in stringency between limits for two different pollutants in the same regulated area, or for the same pollutants across different regulated areas, can influence the technologies they advantage. For example, the difference in the stringency of vehicle emissions standards between the US and EU resulted in divergent market shares of gasoline- and diesel-powered vehicles in the two markets^45,46^. Stricter limits for CO_2_ emissions and more relaxed limits for NO_x_ emissions, coupled with vehicle and fuel taxes which favored diesel, contributed to the pervasiveness of these vehicles in Europe, with negative impacts on air quality^46–48^ (Fig. 1b, f). This was the case because vehicles running on diesel fuel emit less CO_2_ and more NO_x_ compared to vehicles using gasoline. In contrast, stricter air pollution standards inhibited the diffusion of diesel-powered vehicles in the US. A mismatch in regulatory stringency between countries can thus prevent cross-border technology adoption and transfer and may also restrict the international flow of technology patents^49^. Particularly in the EU, incentives, taxes, and standards based on CO_2_ emissions have been shown to favor diesel vehicles, with a trade-off between greenhouse gas emissions mitigation and air pollution abatement goals^47,50,51^.

Rather than requiring absolute compliance for each vehicle, some jurisdictions have incorporated flexibility in the form of market-based compliance credits into performance-based regulation that can be traded between firms, applied across compliance categories, or banked for use in future years. Fuel economy standards, vehicle emissions standards, low-carbon fuel standards, biofuel mandates, and ZEV mandates in the US, Canada, and South Korea all include credit-trading design features^52^. Credit trading offers incentives for technology innovation greater than under straightforward pollution regulation^53^. However, such flexibility may backfire when the regulated technologies produce pollution that is concentrated locally rather than well-mixed in the atmosphere or environment.

Another form of compliance flexibility is the separate categorization of larger vehicles for compliance with fuel economy standards in the US. Under the US CAFE standard, light trucks are subject to more relaxed fuel economy requirements, which limited improvements to their fuel economy from 1978 to 2018^54^. These decades coincided with a growing market share of SUVs, restricting the climate and air quality benefits of the CAFE standard. In contrast, Chinese standards for fuel consumption in passenger cars, adopted in 2004, resulted in fuel economy improvements even in heavier passenger vehicle classes^55^. Crucially, every vehicle sold at the time had to meet the standard for its weight category, meaning that Chinese manufacturers could not use sales of smaller or more efficient vehicles to compensate for a lack of compliance in larger or less efficient vehicles^56^.

Technology-specific policy instruments offer targeted support that helps disruptive technologies overcome the numerous technical, regulatory, and market barriers to their commercialization. However, there are risks and uncertainties associated with selecting a particular technology at the expense of other technologies in less advanced stages of development. Mandating or subsidizing a technology without providing strong incentives for its continued technical and environmental improvement could lead to technological stagnation and lock-in, as illustrated by the US Renewable Fuel Standard which has contributed to a persistence of first-generation biofuels and constrained technical variation and progress in advanced biofuels^57^.

Both regulatory and economic policy instruments (e.g., vehicle emissions standards, vehicle subsidies, etc.) can be attribute-based, meaning that the stringency of the regulation or amount of the subsidy depends on a secondary characteristic like vehicle weight, size, or footprint^58^. Under the US CAFE standard, where vehicle fuel economy requirements vary with vehicle footprint, manufacturers of larger vehicles are subject to lower fleet-wide fuel economy standards than those producing smaller vehicles and are incentivized to increase vehicle size to face a lower standard for compliance^59–62^. Likewise, in Japan, weight-based fuel economy regulation has allowed firms producing heavier cars to face relaxed fuel economy requirements, causing the average weight of Japanese vehicles to increase. This trend has reduced the fuel savings from the regulation and exacerbated traffic accidents as heavier cars pose a greater hazard to non-occupants^58^. EU weight-based emissions standards similarly limit the achievable emissions reductions relative to a flat standard, under which automakers would have otherwise adopted low-carbon technologies or sold smaller cars^41^. In these ways, attribute-basing weakens the stringency and reduces the effectiveness of regulations, sometimes resulting in negative greenhouse gas emissions outcomes (Fig. 1a, b).

Fuel economy standards, taxes, and subsidies are non-linear when the stringency or level of the requirement or incentive changes depending on which side of the threshold the targeted vehicle falls^63^. These policy instruments are said to contain notches as their requirements are non-linear. In China, non-linear fuel economy standards have caused distortions in vehicle design, with manufacturers making modifications to vehicle models in order to face less stringent regulatory requirements^64^. Evidence from Japan suggests that fuel economy incentive schemes with non-linear schedules deterred technology innovation as automakers exaggerated reported fuel economy for vehicles close to eligibility thresholds^65^. When combined with attribute-basing, non-linearity further disincentivizes light-weighting compliance strategies by car manufacturers and leads to forgone fuel economy improvements^41,58,66^.

### Policy instrument choice

**Fig. 3:**
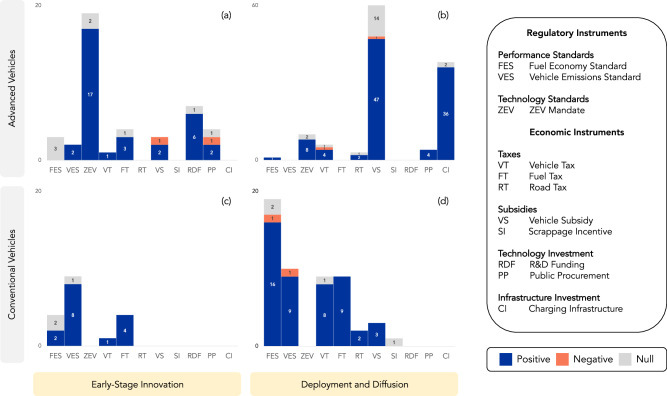
Ex post evaluations of policy instruments’ early-stage innovation vs deployment and diffusion outcomes for conventional vs advanced vehicle technologies. The number of ex post evaluations, measured on the vertical axis, for “Early-Stage Innovation” outcomes (RD, PA) of road transportation policy instruments are shown on the left side of the figure (**a**, **c**). “Deployment and Diffusion” outcomes (MI, FE, SA, TT, CR) appear on the right side (**b**, **d**) (see Supplementary Table 2 for outcome definitions). Evaluations associated with advanced vehicles are shown in the top half of the figure and conventional vehicles in the bottom half. Policy instruments are grouped according to Table 1 and are defined in Supplementary Table 1. Positive-impact evaluations are shown in blue, negative in orange, and null in gray.

**Fig. 4:**
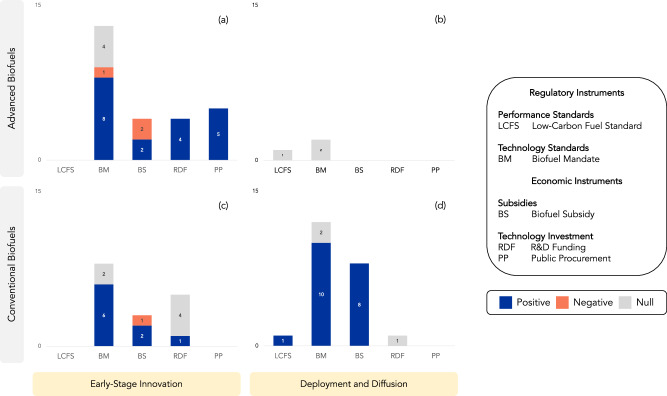
Ex post evaluations of policy instruments’ early-stage innovation vs deployment and diffusion outcomes for conventional  vs advancedbiofuel technologies. The number of ex post evaluations, measured on the vertical axis, for “Early-Stage Innovation” outcomes (RD, PA) of road transportation policy instruments are shown on the left side of the figure (**a**, **c**). “Deployment and Diffusion” outcomes (BP, TT, CR) appear on the right side (**b**, **d**) (see Supplementary Table 2 for outcome definitions). Evaluations associated with advanced biofuels are shown in the top half of the figure and conventional biofuels in the bottom half. Policy instruments are grouped according to Table 1 and are defined in Supplementary Table 1. Positive impacts are shown in blue, negative in orange, and null in gray.

The impact of different types of road transportation policy instruments on innovation varies depending on the stage of innovation and type of technology (Figs. 3 and 4). In Figs. 3 and 4, we have divided policy instrument evaluations along these dimensions, with early-stage innovation indicators on the left and deployment and diffusion indicators on the right, conventional technologies in the lower half of the figure and advanced technologies in the upper half. Early-stage innovation is associated with laboratory experimentation and demonstration, while deployment and diffusion are characterized by learning and cost reductions, which occur as technologies are more widely adopted and new industries grow.

Government R&D funding, public procurement, and support for demonstration have typically underpinned emerging technologies, though for vehicle technologies we find that regulatory policy instruments also have positive impacts on early-stage innovation outcomes (i.e., R&D spending, patents, publications, and prototypes). Fuel economy and vehicle emissions standards tend to be associated with incremental improvements in conventional (internal combustion engine) vehicles, and the ZEV mandate, a type of technology standard, has the most evidence in early-stage innovation outcomes for advanced (electric) vehicle technologies. In general, we find that economic policy instruments have driven deployment and diffusion in both conventional and advanced vehicle technology categories, though fuel taxes stimulate early-stage innovation in gasoline and diesel cars as well as in EVs^23–26^ (Fig. 3a, c).

As electric vehicle technologies diffuse, some policy instruments experience diminishing effectiveness. In particular, support for charging infrastructure is more effective in the earlier stages of EV deployment due to the relative price insensitivity of early adopters and the diminishing marginal benefits of charging infrastructure as the number of charging stations reaches saturation in more mature markets^67–69^. Overall, charging infrastructure availability, density, and speed have been shown to strongly and specifically increase the sale of EVs (Fig. 3b).

Although there are studies linking policy instruments to deployment, and deployment to cost reductions, there is scarce evidence directly attributing cost reductions to specific policy instruments (CR in Fig. 1). Before 2010, declines seen in the cost of lithium-ion cells, which are used in electric vehicles and portable electronic devices as well as in other applications, were not driven by EV deployment.

Several policy instruments have stimulated early-stage innovation in advanced biofuels (Fig. 4a), but this has not translated into the production of these fuels at scale^57,70–72^ (Fig. 4b). Biofuel subsidies have increased the production of conventional (i.e., first-generation) biofuels at the expense of the development of advanced biofuels^40,43^ (BS in Fig. 4a). This is problematic as the framing by policymakers and industry groups of conventional biofuel mandates and subsidies as tools to reduce emissions in road transportation is not substantiated by ex post evidence from the academic literature (Fig. 1e). Although low-carbon fuel standards reward incremental improvements in life-cycle fuel carbon intensity, they have also not resulted in the commercial production of cellulosic biofuels^72,73^ (Fig. 4b). See Supplementary Information SI.2 for further discussion on the environmental outcomes of policy instruments associated with biofuels.

### Policy instrument sequencing

**Fig. 5:**
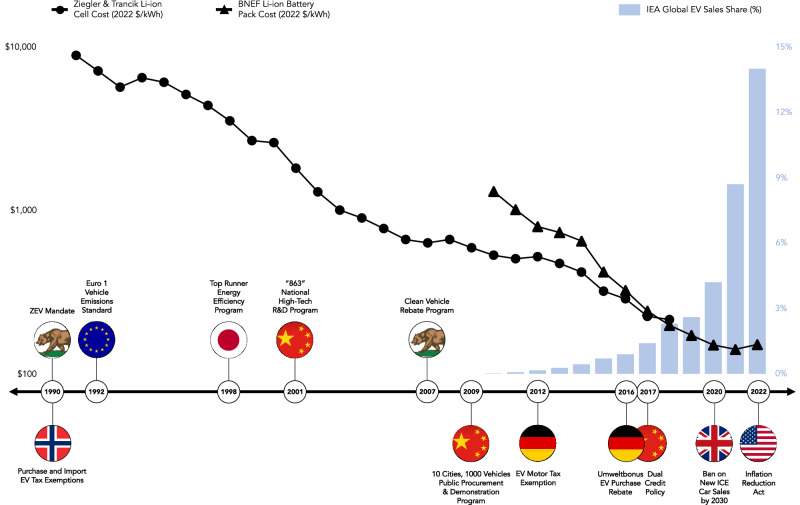
Timeline of selected policy milestones in the evolution of electric vehicles. Source: own elaboration, modeled after Nemet (2019)^103^, using cost and sales data from BNEF, IEA, and Ziegler and Trancik (2021)^104,105^.

Policy instrument sequences where green industrial policy is followed by stringent regulation and pricing have been shown to nurture economic interest groups that support sustainability transitions in the electricity sector^74^. Rather than imposing costs directly on polluters, a “benefits-to-costs” policy sequence initially emphasizes, inter alia, government funding and economic incentives for R&D, manufacturing, and technology deployment. However, in the road transport sector, we find that several important jurisdictions did not follow this pattern; there are also clear differences in policy instrument sequencing between regions. In particular, the sequencing of policy instruments in California in the context of electric vehicles differs from policy instrument sequences seen in China and Norway, global leaders in EV manufacturing and adoption, respectively. While China pursued an industrial policy approach to EVs that initially used grants, subsidies, and public procurement (Fig. 5) to promote domestic innovation and competitiveness, California’s technology-forcing Zero-Emission Vehicle Mandate was introduced in 1990 as a minor provision within a larger vehicle emissions reduction and clean fuels program^75^, largely predating the formation of supportive economic and industrial coalitions. The ZEV mandate was a de facto technology standard that required 10 percent of car manufacturers’ light-duty vehicle sales to be zero-emission by 2003. Although its requirements were suspended and weakened by policymakers in response to lobbying and litigation by automakers, the mandate catalyzed the development of electric drivetrain technologies by start-ups and incumbent firms and facilitated the diffusion of hybrid electric vehicles^76^. In contrast, Norway’s consumer-focused electrification strategy leveraged strong vehicle taxes and upfront subsidies, among other incentives, to encourage electric vehicle sales.

The impacts of policy instruments cannot be isolated to a particular region as technology innovation systems extend beyond these boundaries^77^. Policy instruments implemented in highly industrialized regions like California and Norway have driven technology innovation and adoption in other countries both by influencing corporate innovation strategies and through policy diffusion. In response to evolving regulatory demands shaped by pioneering regions and policies like California’s ZEV mandate, multinational automakers based in Germany and Japan—countries with strong conventional automotive industries—invested in battery EV technologies and made adjustments to their market portfolios^78^. Meanwhile, China’s strategic technology investments and subsidies supporting its EV industry alongside joint ventures with international partners have placed it at the forefront of EV and battery supply chains, benefiting from learning effects in cell manufacturing, pack integration, and increasing economies of scale that drive technology cost reductions^79^. In 2017, China issued the “dual-credit policy” (Fig. 5), a combined zero-emission vehicle mandate and fuel economy standard based partly on California’s ZEV mandate, a policy model that has also proliferated across a number of US states^80^. Motivated by growing concerns over China’s dominance in this sector, recent US policies (Fig. 5) have introduced subsidies for batteries and EVs with local content requirements^81^.

Both California and Norway lacked manufacturing capacity for conventional road vehicles which limited the political and economic influence of incumbent automotive industry groups, enabling the introduction of policies supporting electric vehicle technology innovation and adoption^82^. Though costly, Norway’s EV policies faced relatively little opposition as policymakers were less restrained by concerns around the competitiveness of a domestic industry. In California, severe urban air pollution and a history of technology-forcing command-and-control regulation also contributed to the adoption of its ZEV mandate^83^. Policy milestones in the development of EVs over the past three decades suggest that early movers provided strong support for pre-commercial technologies. Later adopters engaged opportunistically to catch up to and leapfrog established competitors, while some governments instituted reactive measures to regain competitive advantage, shaping the technology and policy trajectories of electric vehicles (Fig. 5). Although Fig. 5 highlights policies specific to road transportation and most directly relevant to innovation in electric vehicles, a number of regions have introduced broader policies covering emissions from multiple sectors, such as emissions-trading systems. Three of China’s seven pilot emissions trading programs covered transport at the city level, though its national ETS, introduced in 2021, does not. Meanwhile, California’s cap-and-trade policy, introduced in 2012, has been integral to the state’s broader strategy to reduce greenhouse gas emissions^84^.

Policy instruments that mandate the production or use of pre-commercial technologies face additional obstacles when the technologies they support encounter multiple forms of lock-in. For instance, the mandate and subsidies supporting first-generation biofuels in the US preceded the introduction of the cellulosic mandate component of the US Renewable Fuel Standard, cementing early path dependencies in corn ethanol. Advanced biofuels now compete not only with fossil fuels but also with vehicle electrification and mature first-generation biofuels under a “blend wall,” a production threshold in the US that limits biofuels to about 10 percent of the transportation fuel mix by volume, unless there is greater adoption of flex-fuel vehicles. By design, biofuel mandates have generally treated biofuels as a complement to fossil fuels rather than a replacement. Moreover, unrealistic goals set by biofuel companies for cellulosic biofuels, which use non-food feedstocks such as crop and forest residues, ultimately led US policymakers to weaken, delay, and simply waive the cellulosic component of federal biofuel mandates altogether, creating uncertainty for producers and investors^85^. Unlike California’s ZEV mandate, which helped commercialize hybrid electric vehicles that share technical similarities with fully battery electric vehicles, the US Renewable Fuel Standard initially focused only on the production first-generation biofuels, which are based on food crops like corn and require different technological and infrastructure investments than second- and third-generation biofuels due to disparities in feedstock and production processes.

## Discussion

We have reviewed the technology innovation and environmental outcomes of 14 road transportation policy instruments from more than 1000 evaluations across 468 studies. We analyzed the impact of policy instrument design, interactions, choice, and sequencing on these outcomes and explored synergies and trade-offs between policy instruments.

We identify and describe six types of road transportation policy instrument interactions: pre-condition, synergy, overlap, addition, trade-off, and backfire. The literature reviewed as a whole indicates that multiple policy instruments in concert can be more effective for promoting technology innovation and environmental sustainability in road transportation than single instruments alone. It is possible for a mix of policy instruments to act together to achieve the levels of stringency necessary to meet desired targets without placing the burden of doing so on a single instrument, while increasing public acceptance, political feasibility, and the potential for successful implementation^16^. Some policy instruments can be successful only in combination with or subsequent to the implementation of another instrument, as illustrated by electric vehicle subsidies and support for charging infrastructure in the earliest stages of EV deployment. However, additional policy instruments sometimes trade off or backfire: Alongside binding biofuel mandates, subsidies for biofuel production increase greenhouse gas emissions by lowering fuel prices, and electric vehicle subsidies and mandates trade off with fleet-wide internal combustion engine vehicle performance standards by allowing automakers to comply with the latter using weaker improvements in conventional vehicle emissions and fuel efficiency. Reducing cross-compliance between policy instruments targeting conventional as opposed to advanced technologies could allow larger improvements in both categories.

Design features that discourage vehicle size and weight reduction are undesirable as limiting vehicle weight is a vital lever for reducing the emissions of road vehicles^86^. Among the policy instrument design features we identify and classify, we find that compliance flexibility, attribute-basing, and non-linearity in fuel economy standards, taxes, and subsidies have favored sport-utility and other large vehicles, which use more fuel and generate more air pollution and greenhouse gas emissions than passenger cars. Carve-outs for these vehicles in US fuel economy standards and weight-based vehicle regulations in Japan have also incentivized manufacturers to increase vehicle size and weight in order to avoid more stringent compliance levels, taking advantage of the improved economics of manufacturing, advertising, and selling SUVs enabled by these design features. Attribute-basing on driving range, battery capacity, or vehicle size in electric vehicle subsidies could similarly influence automakers’ EV production strategies, with possible environmental and performance trade-offs. EVs are already commonly subsidized according to their battery capacity, with higher battery capacities eligible for larger subsidies, an approach which increases the sale of EVs^87^ but could also increase vehicle life-cycle emissions relative to a flat subsidy^88^. This is because in electric vehicles, battery capacity is correlated with vehicle mass and energy consumption, and smaller battery packs offer more emissions benefit for each dollar spent because of charging requirements and other factors^89^. Although compliance flexibility, non-linearity, and attribute-basing in policy instruments may increase their acceptance among automakers, they also discourage vehicle size and weight reduction and are therefore counterproductive to sustainability objectives. Performance standards and subsidies for vehicles should reduce or avoid the use of such features.

Overall, our analysis highlights the complex task faced by policymakers in balancing different technology and environmental priorities, adapting to technological uncertainty, and managing interference from powerful industry actors, challenges which are experienced in other sectors beyond road transportation (e.g., shipping, aviation, and agri-food production and consumption). In particular, technology-specific policy instruments can lead to early technological lock-in of more mature but flawed alternative technologies, even when the intention is for the technology-ready solution to serve as an intermediate to more advanced solutions. This is most evident in our analysis of the outcomes of conventional biofuel mandates and subsidies, which the evidence suggests are a factor in the lagging development of cellulosic biofuels, an advanced biofuel technology that must overcome the lock-in not only of incumbent fossil fuels but also of mature first-generation biofuels while competing with increasing road vehicle electrification, all of which receive government support. The experience with biofuels to date offers potential lessons for the design of other technology-specific policy instruments, like ZEV mandates, where proactive efforts to manage renewable electricity integration, vehicle energy consumption, and supply chain sustainability are becoming increasingly important as EVs achieve higher market penetration^88,90–92^. Policy instruments with high technology specificity may benefit from clear and responsive revisions in technology selection criteria or performance-based elements that require improvements in a technology’s life-cycle environmental impacts over time.

Policy instruments also interact spatially and temporally in ways that affect technology innovation and environmental outcomes^93^. The California ZEV mandate and Norwegian incentives for electric vehicles, implemented in highly industrialized regions at the top of the EV technology experience curve, have influenced EV technology innovation and deployment as well as the diffusion of policies in other jurisdictions. The evolution of the EV industry has been guided in part by policies implemented in different regions over decades depending on their political economy, domestic priorities, industrial structure, and the level of incumbent industry resistance they face. Coordinating action where local problems, politics, and industry dynamics are conducive to ambitious policies, as they were in California and Norway, could provide early direction for sustainability transitions and help spur the scale-up and commercialization of novel technologies in regions with greater manufacturing capacity, expanding global production and reducing technology costs.

Our work has several limitations. First, we have focused on published, peer-reviewed findings from the academic literature, which may differ from results that are unpublished or available in the gray literature. Second, our review includes English-language articles only, as is common in large-scale systematic reviews in environmental and energy policy research. The global scope of our analysis made incorporating studies in other languages infeasible and may have resulted in the exclusion of potentially relevant sources of evidence. Third, our analysis of policy instruments has focused on metrics of innovation and their associated environmental outcomes, but it is important that policy instruments reducing travel demand, encouraging modal shifts, promoting more sustainable transport and land use planning, altering driving behavior, and phasing out polluting technologies are not neglected in work on road transportation sustainability. We have also not reviewed public health, social, or distributional outcomes, which may mediate the effectiveness of road transportation policy instruments and are worth investigating in their own right. We believe these are useful areas for additional analysis and synthesis. Fourth, given the disparate nature of the evidence and the myriad methodologies producing it, we did not seek to directly compare the effectiveness of policy instruments nor analyze magnitudes of effect sizes for different outcomes. Instead, we take a broad sectoral view of the innovation process across multiple stages to identify synergies and trade-offs associated with road transportation policy instruments in different stages of technology innovation. Our analysis does not suggest that all applications of road transportation policy instruments would yield outcomes identical to those covered here—as we have demonstrated, different political contexts, industrial structures, policy design choices (e.g., stringency, flexibility, specificity, non-linearity, etc.), interactions with other policy instruments, timing, and sequencing can and do affect the direction of technology innovation and environmental outcomes.

Although our systematic review includes some studies on heavy-duty road vehicles, the evidence comes largely from private passenger transport, which mainly involves light-duty vehicles. Future work investigating the impact of policies on innovation in heavy-duty vehicle technologies could help inform mitigation actions for road freight, which contributes substantially to road transportation CO_2_ emissions as well as to air pollution and is likely to require government intervention to transform, whether through emissions and performance standards or incentives for technology development, adoption, or demand reduction. Another important yet neglected area of research and regulation has to do with non-exhaust emissions, or particulate emissions from break, tire, and road wear rather than from fuel combustion^94^, which make up an increasing proportion of total road vehicle emissions, in part due to the increasing regulation of tailpipe emissions. The adoption of technologies to reduce non-exhaust emissions faces similar hurdles to the adoption of tailpipe emissions control technologies, though the technologies themselves differ substantially. Ex ante research on policy instruments for the abatement of non-exhaust emissions could help fill this regulatory gap by identifying how and where interventions may be most effective in reducing this form of pollution from road transportation.

Research attributing and quantifying the impact of specific transportation policy instruments or mixes on technology cost reductions—for example, in batteries, electric vehicles, and alternative fuels—could improve the accuracy of technology cost forecasting and integrated assessment and energy-economic scenario modeling for this sector, particularly in the context of decarbonization in both national-level and global analysis. In addition, evidence on the impacts of transportation policy instruments on technology innovation and adoption in emerging economies, and particularly on technology transfer, remains limited, as is indicated by this review. Thus, further work assessing these impacts, with attention to trade-offs and negative externalities, could be insightful to policymakers focused on technology cooperation and competition as they relate to sustainability transitions given the importance of technology transfer in reducing the environmental impacts of transportation. Finally, few studies evaluate the effects of policy instruments covering multiple sectors, such as carbon taxes and emissions-trading systems, on technology innovation, or compare these with sector-specific policy instruments, an area of research we believe would help clarify the relative importance of such instruments for sustainability transitions, particularly as carbon prices increase, which is likely to increase their impact.

## Methods

### Systematic review framework

We follow a systematic approach in six stages to extract, analyze, and synthesize evidence from the academic literature on the technology innovation and environmental outcomes of road transportation policy instruments. The data we use were identified, collected, screened, and analyzed using the Web of Science Core Collection, Mendeley Reference Manager 2.100.0 and Microsoft Excel 16.60.

Our analysis focuses on two broad categories of public policy instruments: regulatory and economic (Table 1). We further differentiate policy instruments into six classes and, within these, define 14 types of individual policy instruments (see Supplementary Table 1 for policy instrument definitions). Though we have included many of the most common road transportation policy instruments across a variety of geographical, technological, and temporal contexts, we have not included voluntary emissions reduction agreements in this review as previous evidence has indicated that their lack of stringency and enforceability provide weaker incentives for technology innovation compared to mandatory-compliance policy instruments^35,95–98^. Soft instruments such as fuel economy labels, preferential parking or lane access, and other non-monetary incentives are also outside the scope of this review.

Indicator-based assessments are frequently used to evaluate the effectiveness of environmental and energy policy instruments in terms of their impact on technology innovation^12,14,99^. Because we are interested in the ability of policy instruments to stimulate innovation in both conventional and advanced vehicle and biofuel technologies for road transportation sustainability, we include indicators representing both technology innovation and environmental outcomes for evaluating policy instruments in the systematic review. A preliminary evaluation framework was developed a priori and adjusted in response to several initial searches.

Adapting existing frameworks^12,14,100^ for energy technology innovation, we divide indicators into four groups: innovation inputs, innovation outputs, innovation outcomes, and environmental outcomes (Table 2; see Supplementary Table 2 for definitions). We refer to all of these collectively as “outcomes.” Innovation inputs include financial inputs to the innovation process, typically reported in our literature sample as R&D spending (RD in Fig. 1) by private or semi-private companies. Innovation outputs include products of the R&D process such as patents, publications, and prototypes (PA in Fig. 1). Innovation outcomes include modular improvements (MI in Fig. 1) to vehicles and changes to fuel economy (FE in Fig. 1) as well as other measures of technology diffusion, such as sales of zero-emission vehicles (SA in Fig. 1), biofuel production volumes (BP in Fig. 1), technology transfer (TT in Fig. 1), and technology cost reductions (CR in Fig. 1). The environmental outcomes we consider are greenhouse gas emissions (GG in Fig. 1), air pollution (AP in Fig. 1), and land use (LU in Fig. 1).

### Search strategy

After we define the policy instruments and outcomes of interest, we proceed with the design of the search strategy. In our search, we include peer-reviewed studies from the academic literature that investigate the effects of policy instruments in various countries, time periods, and combinations with other policy instruments. To conduct the search, we use the Web of Science database to search for original English-language peer-reviewed studies from the academic literature published between January 1, 1975 and October 31, 2022. Based on our delineation of the stages of the innovation process and corresponding innovation and environmental indicators, we structure one search string to include common terms and synonyms for each policy instrument and indicator. The IEA transport sector policies database was consulted to determine common naming conventions of selected policy instruments (e.g., renewable fuel standard vs biofuel blend mandate, fuel economy vs fuel efficiency standard) for inclusion in the search string (see Supplementary Fig. 1 for search string details).

Our initial search did not include restrictions on research areas, academic disciplines, or journals. In order to check more robustly that this search string was not systematically excluding papers of relevance, a second search was conducted targeting top transportation journals. This yielded one additional paper that met both our inclusion and exclusion criteria, which was added to the final review sample (see Supplementary Tables 3 and 4 for more details).

### Inclusion criteria

We use the CIMO framework^101^ to specify study inclusion criteria for the search: context (C), intervention (I), mechanism (M) and outcome (O).

#### Context

Policy instruments which relate to road transportation are considered. Studies assessing the impacts of policy instruments on maritime, rail, or air transport technology innovation or environmental outcomes are therefore not included. Terms in the search strings representing the context of our search include “transport”, “road”, “vehicle”, and “fuel”. We did not make any restrictions to the search based on geographical location.

#### Intervention

Sector-specific road transportation policy are considered. Intervention search terms incorporate common naming conventions for policy instruments in the review and include, for example, “fuel economy”, “energy performance”, “low-carbon fuel”, “sustainable fuel”, etc., coupled with terms describing types of policy instruments such as “regulation”, “subsidy”, “mandate”, “tax”, etc.

#### Mechanism

Studies that evaluate or model the effect of a specific policy instrument or combination of instruments on an innovation or environmental outcome are included. In the search string, we use “impact”, “outcome”, “estimate”, “evidence”, “evaluation”, etc.

#### Outcome

Studies that measure, model, or qualify the impact of a single policy instrument or set of policy instruments on the selected technology innovation and environmental outcomes are included. We include terms representing different stages and types of innovation and environmental outcomes like “invention”, “cost reduction”, “market penetration”, “air pollution”, “land use”, “emissions”, etc.

We include both ex ante and ex post evaluations in the systematic review. Including evaluations from ex ante modeling studies enables us to examine the mechanisms through which policy instruments, their design, and interactions produce different innovation and environmental outcomes. See Supplementary Fig. 1 for the full systematic review search string which was used to identify relevant studies.

### Exclusion criteria

Once the inclusion criteria are defined based on the CIMO framework, the definition of exclusion criteria allows us to further focus and reduce the number of studies to those that evaluate the impacts of road transportation policy instruments (Table 1) on the technology innovation and environmental outcomes (Table 2) of interest. First, studies where the type of policy instrument or outcome was not clearly defined (e.g., those evaluating a broad category of instruments but not specifying any concrete type of policy instrument) are excluded from the final sample. Second, studies where the policy instruments making up a policy mix could not be distinguished are excluded (i.e., studies evaluating policy portfolios or packages that do not identify specific policy instruments). Third, studies focusing on the environmental outcomes of a technology without reference to a specific policy instrument are excluded. Fourth, studies where the only benchmark of comparison is another policy instrument are excluded. Lastly, voluntary agreements, local-level policy instruments focusing on traffic and congestion (e.g., low-emissions zones), and general economy-wide policies (e.g., carbon pricing covering multiple sectors) are excluded. We are not able to cover all policies in this review: One reason for our focus on road transportation-specific policy instruments is that because different sectors are at various stages of sustainability transitions, encompass different technologies, and have different political contexts, the challenges that policymakers must address are diverse and context-specific and thus effective policies for socio-technical transitions are often sector-specific^8^. In addition, general carbon pricing strategies tend to neglect specific sources of lock-in and opportunities for innovation as they are not tailored to sectoral contexts^102^, and previous work focused on technologies for the decarbonization of the power sector has suggested that sector-specific policies have relatively larger impacts on technology innovation^103^.

### Screening

Our initial search returned 18,335 papers. After removing duplicates, applying the inclusion and exclusion criteria for titles, abstracts, and the main text to these papers, as well as including additional studies from outside the initial search results, a final set of 468 peer-reviewed studies comprising more than 1000 evaluations on the impacts of road transportation policy instruments on the selected innovation and environmental outcomes were included in the final literature sample (see Supplementary Table 3 for details on each stage of the screening process). We note that some studies evaluate multiple policy instruments, hence the higher number of total evaluations than unique studies, and there is some overlap between policy instrument groups, for example, between vehicle taxes and vehicle purchase subsidies. For ex post analyses, we include studies where the empirical study design supports the interpretation of a credible link between an existing or historical policy instrument and the outcome. Studies linking production volume or deployment levels with cost reductions without attempting to account for the influence of relevant policy instruments, are excluded from the systematic review. For ex ante analyses, we include studies that investigate the effects of a hypothetical policy instrument or the future effects of an existing policy instrument rather than the effects of a specific technology in isolation. For instance, some consequential life cycle assessments (LCAs) are eligible for inclusion but attributional LCAs, which focus on the impact of specific technologies, are not.

### Coding

Policy instrument evaluations were coded positive (negative) if the outcome was considered desirable (undesirable) in the context of the policy implemented. For example, policy instruments associated with increased greenhouse gas emissions, air pollution, or land use were coded negative for these outcomes. A null outcome indicates that the study did not identify a connection between the policy instrument and relevant technology innovation or environmental outcome, or did not find a change in the observed or modeled outcome. We caveat that a positive or negative outcome does not imply an absolute positive or negative outcome globally, and outcomes should not be considered equivalent in magnitude. We have also coded and ordered the strength of the evidence based on its methodology using the classification in Supplementary Table 5. However, we do not weigh specific evaluations in our analysis given the wide-ranging scope of our research, the disparate outcomes and measures, and the variety of both qualitative and quantitative evidence reviewed (see Supplementary Data 2 for the final list of papers included in the systematic review, ordered by strength of evidence). A subset of studies was double-coded to check consistency in coding between researchers.

### Reporting summary

Further information on research design is available in the Nature Portfolio Reporting Summary linked to this article.

## Supplementary information

Supplementary Information

Description of Additional Supplementary Files

Supplementary Data 1

Supplementary Data 2

Supplementary Data 3

Reporting Summary

Peer Review file

## Data Availability

The data analyzed in this study are available as Supplementary Information.
