# Supplementary material for: Technology innovation and environmental outcomes of road transportation policy instruments
Source: Nat Commun. 2025 May 14;16:4467. doi: 10.1038/s41467-025-59111-8 (PMC12078554; doi:10.1038/s41467-025-59111-8)
Supplement: Supplementary file 1 — Supplementary Information [file 41467_2025_59111_MOESM1_ESM.pdf]

Technology innovation and environmental outcomes of road transportation policy instruments

Clara Ma[1 2], Cristina Peñasco[1 3 4], Laura Díaz Anadón[1 2 5]

[1]Centre for Environment, Energy and Natural Resource Governance, Department of Land Economy, University of Cambridge, Cambridge, UK
[2]Conservation Research Institute, University of Cambridge, Cambridge, UK
[3]Department of Politics and International Studies, University of Cambridge, Cambridge, UK
[4]Centre for Climate and Nature, Banque de France, Paris, France
[5]Belfer Center for Science and International Affairs, Harvard Kennedy School, Harvard University, Cambridge, MA, USA

Supplementary Information

SI.1: Supplementary Methods

Supplementary Fig. 1: Systematic review search string

CONTEXT	transport* OR vehicle* OR *fuel* OR automobile OR automotive OR road OR car OR bus OR truck OR battery OR engine OR motor*
INTERVENTION	((“fuel economy" OR "fuel efficiency" OR "fuel consumption" OR "energy efficiency" OR "energy performance" OR emission* OR "renewable fuel" OR low*carbon*fuel OR "sustainable fuel" OR "alternative fuel" OR "fuel quality" OR “clean fuel” OR biofuel) NEAR/1 (standard OR limit OR regulation OR requirement OR obligation OR mandate OR directive OR program)) OR (tax OR credit OR incentive OR rebate OR fee OR subsidy OR bonus OR malus OR discount) OR ((public OR government) NEAR/1 (procurement OR demonstration)) OR “charging infrastructure” OR ((“research and development” OR R&D OR RD&D) NEAR/1 (funding OR investment OR subsidy OR program OR expenditure* OR spending))
MECHANISM	impact* OR effect* OR outcome* OR estimate OR evidence OR evaluation OR assessment OR analysis OR appraisal OR model OR projection OR simulation
INNOVATION OUTCOMES	invention OR innovation OR development OR demonstration OR deployment OR commerciali*ation OR “market formation” OR adoption OR diffusion OR “market share” OR “market penetration” OR (techn* NEAR/1 (change OR improvement OR progress)) OR patent* OR publication* OR bibliometric OR prototype* OR sale* OR "technology transfer" OR spillover OR "cost reduction" OR “learning curve” OR “experience curve” OR learning*by*doing OR "Porter hypothesis"
ENVIRONMENTAL OUTCOMES	environment* OR (air NEAR/1 (pollution OR quality)) OR “land use” or emission*

Supplementary Table 1: Definitions of policy instruments included in systematic review

Policy Instrument	Definition
1. Regulatory Instruments	
1.1 Performance Standards	
Fuel Economy Standard (FES)	Regulation setting minimum requirements for fuel efficiency of vehicles sold within a particular market, applied either to individual vehicles or to a fleet average
Vehicle Emissions Standard (VES)	Regulation limiting emissions of CO_2 or air pollutants (e.g., NO_x, CO, PM) from vehicles
Low-Carbon Fuel Standard (LCFS)	Regulation requiring reduction in carbon intensity of transportation fuels, setting a limit on the amount of CO_2 and other greenhouse gases that can be emitted during the production and use of fuels throughout its life cycle
1.2 Technology Standards	
Zero-Emission Vehicle Mandate (ZEV)	Requirement for vehicle manufacturers to sell a certain share of zero- or low-emission vehicles, typically battery electric vehicles or plug-in hybrids
Biofuel Mandate (BM)	Requirement for fuel suppliers to blend a specific share or volume of biofuels into transportation fuels
2. Economic Instruments	
2.1 Taxes	
Vehicle Tax (VT)	One-time fee paid at time of vehicle purchase or registration, often based on vehicle characteristics such as value, weight, horsepower, engine displacement, fuel efficiency, CO_2 emissions, etc.
Road Tax (RT)	Fee paid annually by vehicle owners using public roads based on vehicle characteristics such as value, weight, horsepower, engine displacement, fuel efficiency, CO_2 emissions, etc.
Fuel Tax (FT)	Government-imposed charge on the sale of transportation fuel, usually calculated per liter or per gallon
2.2 Subsidies	
Vehicle Subsidy (VS)	Financial incentive provided by governments to reduce the cost of purchasing or operating low- or zero-emission vehicles, typically aimed at encouraging the adoption of battery electric, plug-in hybrid or other fuel-efficient vehicles; can take various forms, such as tax credits or exemptions, rebates, grants or discounts, with varying eligibility requirements
Scrappage Incentive (SI)	Government vehicle scrappage or trade-in program encouraging replacement of old, often less fuel-efficient vehicles with newer, more fuel-efficient or lower-emissions models, typically involving financial incentives or discounts to vehicle owners who retire older vehicles and/or trade them in for new, more fuel-efficient models

Biofuel Subsidy (BS)	Financial incentive provided by governments to fuel producers or blenders to promote production of biofuels; can take various forms, such as tax credits or exemptions, grants, etc.
2.3 Technology Investment	
R&D Funding (RDF)	Public funding, grants, subsidies for, or direct investments in emissions- or fuel-saving vehicle and fuel technology research and development
Public Procurement and Demonstration (PP)	Government purchase – or funding of projects to demonstrate viability – of vehicle and fuel technologies with reduced environmental impact (e.g. electric buses)
2.4 Infrastructure Investment	
Charging Infrastructure (CI)	Government grants, incentives, direct investments, or other programs to support the development, expansion, density, speed or accessibility of electric vehicle charging stations

Supplementary Table 2: Definitions of indicators for policy instrument evaluation

Indicator	Definition
1. Innovation Outcomes	
1.1 Inputs	
R&D Spending (RD)	Financial resources allocated by organizations to conduct research and development aimed at advancing scientific knowledge, creating new technologies, or improving existing products, processes, or services related to reducing the energy consumption or environmental impact of vehicle and fuel technologies
1.2 Outputs	
Patents, Publications, and Prototypes (PA)	Number of patents granted, volume of research publications concerning, or development of functional prototypes of new fuel- or emissions-saving vehicle or fuel technologies or processes
1.3 Outcomes	
Modular Improvements (MI)	Additions of novel fuel- or emissions-saving components (e.g., catalytic converters) to or design changes and improvements in existing components (e.g., engine, transmission, sensors) of conventional (i.e., internal combustion engine) vehicles which do not require alternative vehicle architectures
Fuel Economy (FE)	A measure of the average distance traveled per unit of fuel consumed by a vehicle or fleet of vehicles (typically sales-weighted) for a manufacturer, group of manufacturers, or in a particular market area
Vehicle Sales (SA)	Number or share of low- or zero-emission vehicles sold in a market or region during a specified time period in ex post evaluations; the likelihood or intention of

	purchasing a low- or zero-emission vehicle in ex ante evaluations
Biofuel Production (BP)	Quantity of biofuels produced within a given time period and region
Technology Transfer (TT)	The sharing and disseminating of technology or knowledge as indicated by patents, exports, or technology adoption across countries
Cost Reductions (CR)	The decrease in the cost of a product, process, or technology over time, driven by learning, innovation, economies of scale, or productivity and efficiency improvements
2. Environmental Outcomes	
Greenhouse Gas Emissions (GG)	Level of greenhouse gas emissions in a given area or emissions performance of a fleet of vehicles (positive impacts denote a decrease in emissions or improved emissions performance, while negative impacts denote an increase in emissions or worse performance)
Air Pollution (AP)	Level of air pollutants such as particulate matter, nitrogen oxides, carbon monoxide, and volatile organic compounds in a given area or air pollutant emissions performance of a fleet of vehicles (positive impacts denote a decrease in air pollution or improved performance while negative impacts denote an increase in emissions or worse performance)
Land Use (LU)	Changes in the allocation and utilization of land resources related to the implementation of road transportation policies (positive impacts denote a decrease in land use, while negative impacts denote an increase)

Supplementary Table 3: Screening results

Screening Stage	Number of Documents	Methodological Step
Initial Search	18,335	Search conducted using Web of Science database according to inclusion criteria, returning 18,335 records; 8 duplicate search records removed
Titles	18,327	18,327 titles screened for relevance, resulting in the removal of 14,577 titles
Abstracts	3,750	2,797 abstracts removed due to conflicts with inclusion and exclusion criteria
Full Text	953	541 studies further excluded after full text screening
Additional Studies	55	55 additional studies satisfying inclusion and exclusion criteria but which did not appear in initial search

		results identified from references in included articles
Transportation Studies	1	1 study identified from a second, targeted search of top transportation journals
Final	468	468 studies included in the final review, comprising over 1000 policy instrument evaluations and assessments

Supplementary Table 4: List of transportation journals included in second search, ranked by 2022 journal impact factor

Rank	Journal
1	Analytic Methods in Accident Research
2	Journal of Public Transportation
3	Transportation Research Part E- Logistics and Transportation Review
4	Transport Reviews
5	Transportation Research Part D- Transport and Environment
6	Transport Policy
7	Transportation Research Part B- Methodological
8	Transportation Research Part A- Policy and Practice
9	Journal of Transport Geography
10	Journal of Air Transport Management
11	Accident Analysis and Prevention
12	Travel Behaviour and Society
13	Research in Transportation Business and Management
14	Transportation Science
15	Maritime Economics & Logistics
16	Transportation
17	European Transportation Research Review
18	Journal of Safety Research
19	Transportation Research Part F- Traffic Psychology and Behaviour
20	International Journal of Sustainable Transportation

Supplementary Table 5: Research method types, evidence strength levels, and number of studies in each category from final review sample

Research Type	Strength of Evidence	Number of Papers
Randomized	Level 1	0
Quasi-Experimental/Observational	Level 2	187
Qualitative	Level 3	32
Ex Ante/Theoretical	Level 4	249

Supplementary Fig. 2: Journals represented in final review sample with more than 10 studies

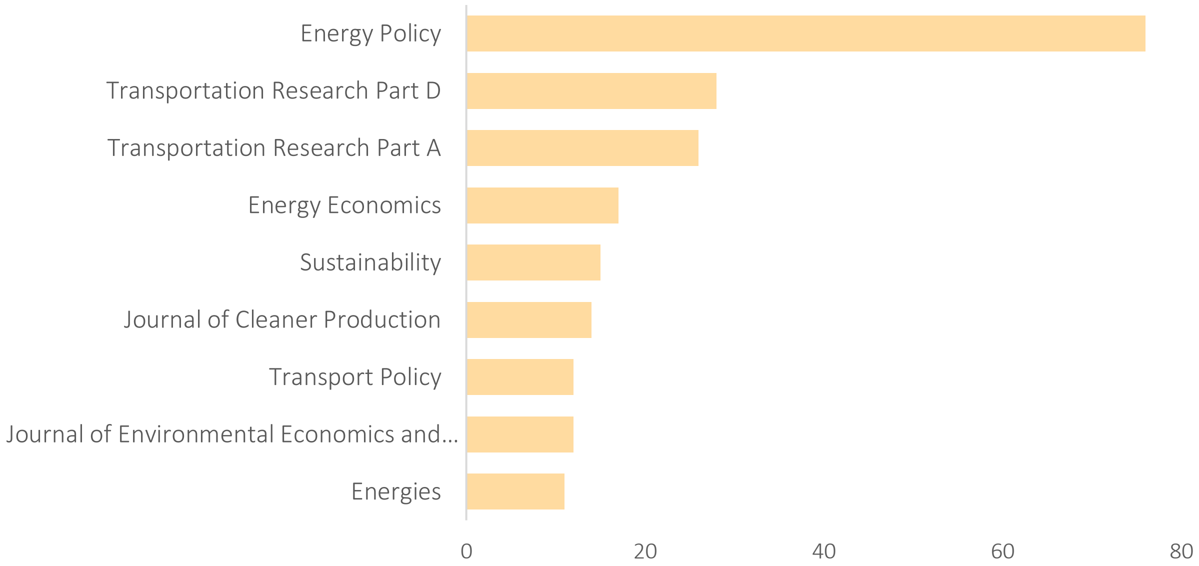

Supplementary Fig. 3: Publication years of studies included in final review sample

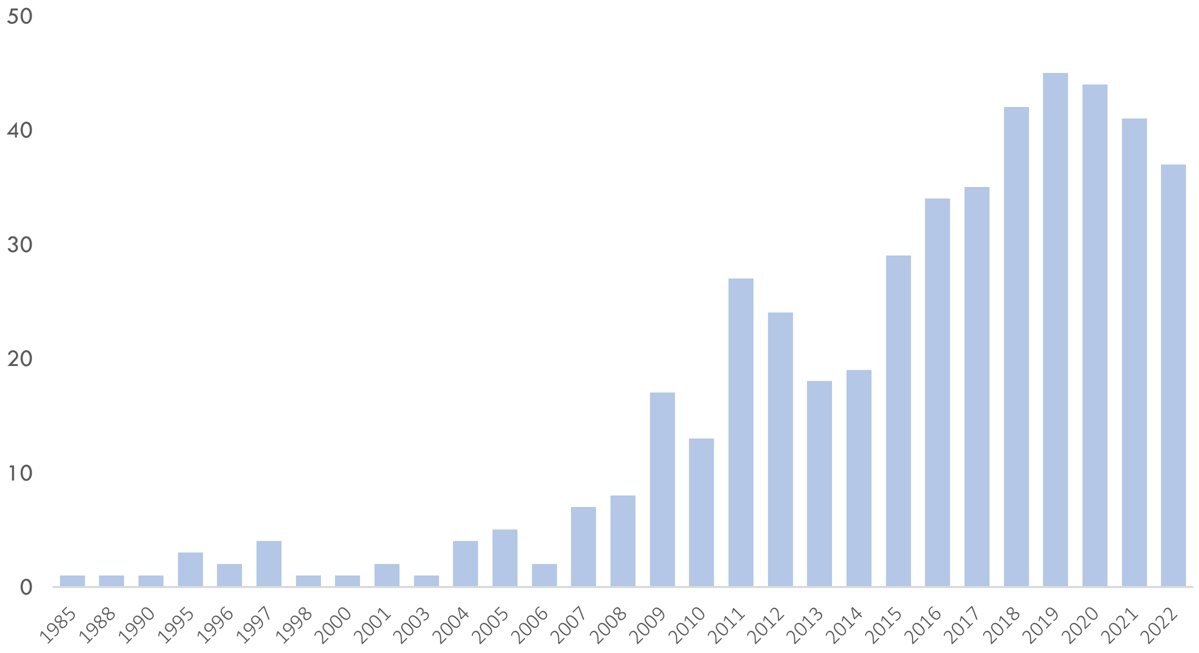

SI.2: Supplementary Discussion

Policy Instrument Design (continued)

Various studies have raised concerns about the distributional impacts of vehicle purchase subsidies, finding that these subsidies favor high-income consumers who are more likely to purchase electric vehicles in the first place, regardless of whether a purchase incentive is available[1–6]. This can weaken the environmental effectiveness of the incentive as low-income consumers are more likely to retire older vehicles with worse emissions performance whereas high-income consumers are more likely to own multiple vehicles and thus purchase additional rather than replacement vehicles[7,8]. Capping the price of eligible vehicles, or the income of eligible consumers, and increasing the size of the incentive for low-income consumers could help improve equity and effectiveness[2,6,8–10], although these caps should be tailored to the stage of market evolution, where early-stage niche markets are composed predominantly of high earners.

Vehicle subsidies may take the form of direct rebates, discounts, tax credits, or tax exemptions. Different forms of subsidies can introduce greater complexity for consumers, which in turn reduces the value the consumer places on the subsidy in the present and weakens the subsidy's overall effectiveness in stimulating vehicle purchases. Greater immediacy and simplicity in obtaining a subsidy substantially enhance the positive impact of the subsidy on low-emissions vehicle sales. Upfront rebates and subsidies applied at the point of sale have had a larger impact on consumer purchase decisions than tax credits, exemptions, and ownership cost reductions[8,11–15]. Sales tax waivers led to 10 times the volume of sales compared to tax credits for hybrid electric vehicle adoption in the US because the former was applied instantly rather than delayed to after the time of income tax filing[11]. To benefit from a tax credit, the buyer must possess a sufficiently large tax burden, which further excludes low-income consumers from eligibility.

Subsidies that do not sufficiently lower the relative vehicle purchase price are not as effective in increasing sales of low-emissions vehicles. For example, monetary incentives for hybrid electric vehicles in the US only shifted consumer purchase decisions when the amount of the subsidy was greater than USD 1000[16].

There is strong agreement that subsidies more effectively induce consumer uptake of EVs when given directly to the consumer rather than to manufacturers or car dealers[3,8,12,17]. However, even when the subsidy is given directly to the consumer, dealers may still be able to adjust the retail price of the vehicle in order to capture part or all of the subsidy[8].

Environmental Impacts and Rebound Effects of Road Transportation Policy Instruments

Because biofuel mandates and subsidies have primarily supported the production and use of first-generation biofuels, they have in some cases resulted in increased greenhouse gas emissions[18–20]. First-generation biofuels use edible feedstocks such as corn, sugarcane, and soy and are associated with negative impacts on land use, water use, water quality, and soil quality[21]. Around half of ex ante evaluations and all ex post evaluations in our review identify

negative greenhouse gas emissions outcomes linked to biofuel mandates (Fig. 1e in the main text). Negative greenhouse gas emissions outcomes of biofuel mandates are driven by emissions from land use change and fertilizer application[20], while the disagreement in the ex ante evidence on greenhouse gas emissions arises from uncertainties in modeling assumptions involving policy attribution, oil prices, technology costs, agricultural yields, indirect land use change, and fuel market rebound effects. For instance, some studies find that mandates could lower the cost of blended gasoline, leading to a rebound in global oil consumption that offsets or negates domestic emissions benefits[22–24]. Others predict positive greenhouse gas emissions outcomes by assuming that the net emissions from biofuel production are zero, neglecting land use change emissions[25]. Ex ante and ex post evidence for biofuel subsidies on greenhouse gas emissions was unanimously negative, with studies finding that biofuel subsidies in the United States effectively subsidized gasoline consumption when combined with a biofuel blend mandate and increased CO2 emissions[26–28]. Biofuel mandates and subsidies are both consistently associated with increased land use and land use change. In contrast to policy instruments promoting biofuels specifically, low-carbon fuel standards, e.g. in the EU, Brazil, California, Quebec, etc., incentivize a broad range of alternative transportation fuels, including renewable electricity, and have been associated with decreasing transportation fuel carbon intensity in some geographic regions[29,30]. Like other policy instruments that are efficiency-promoting or that apply to limited areas, low-carbon fuel standards are prone to rebound effects and leakage. Although fuel economy standards have increased the availability and adoption of vehicles with improved fuel performance, they have caused rebounds in fuel use and substitutions in spending towards other energy-intensive goods and services[31–33]. Similarly, purchase subsidies for fuel-efficient vehicles have in some cases stimulated additional overall vehicle purchases, partially offsetting gains made in pollution abatement, fuel savings, and emissions reductions[34,35]. Combining fuel economy standards with fuel taxes may have the potential to limit rebound effects by reducing the demand for driving while compelling further efficiency gains in the vehicle fleet[36–39].

References

1. Chandra, A., Gulati, S. & Kandlikar, M. Green drivers or free riders? An analysis of tax rebates for hybrid vehicles. *J Environ Econ Manage* **60**, 78–93 (2010).
2. DeShazo, J. R., Sheldon, T. L. & Carson, R. T. Designing policy incentives for cleaner technologies: Lessons from California's plug-in electric vehicle rebate program. *J Environ Econ Manage* **84**, 18–43 (2017).
3. Diamond, D. The impact of government incentives for hybrid-electric vehicles: Evidence from US states. *Energy Policy* **37**, 972–983 (2009).
4. Guo, S. & Kontou, E. Disparities and equity issues in electric vehicles rebate allocation. *Energy Policy* **154**, 112291 (2021).
5. Ku, A. L. & Graham, J. D. Is California's Electric Vehicle Rebate Regressive? A Distributional Analysis. *J Benefit Cost Anal* **13**, 1–19 (2022).
6. Xing, J., Leard, B. & Li, S. What does an electric vehicle replace? *J Environ Econ Manage* **107**, 102432 (2021).
7. Caulfield, B., Furszyfer, D., Stefaniec, A. & Foley, A. Measuring the equity impacts of government subsidies for electric vehicles. *Energy* **248**, 123588 (2022).
8. DeShazo, J. R. Improving Incentives for Clean Vehicle Purchases in the United States: Challenges and Opportunities. *Rev Environ Econ Policy* **10**, 149–165 (2016).
9. Ju, Y., Cushing, L. J. & Morello-Frosch, R. An equity analysis of clean vehicle rebate programs in California. *Clim Change* **162**, 2087–2105 (2020).
10. Sheldon, T. L. & Dua, R. Effectiveness of China's plug-in electric vehicle subsidy. *Energy Econ* **88**, 104773 (2020).
11. Gallagher, K. S. & Muehlegger, E. Giving green to get green? Incentives and consumer adoption of hybrid vehicle technology. *J Environ Econ Manage* **61**, 1–15 (2011).
12. Gong, S., Ardeshiri, A. & Hossein Rashidi, T. Impact of government incentives on the market penetration of electric vehicles in Australia. *Transp Res D Transp Environ* **83**, 102353 (2020).
13. Whitehead, Washington & Franklin. The Impact of Different Incentive Policies on Hybrid Electric Vehicle Demand and Price: An International Comparison. *World Electric Vehicle Journal* **10**, 20 (2019).
14. Fluchs, S. The diffusion of electric mobility in the European Union and beyond. *Transp Res D Transp Environ* **86**, 102462 (2020).
15. Urrutia-Mosquera, J. & Fábrega, J. Impact of fiscal incentives in the consumption of low emission vehicles. *Case Stud Transp Policy* **9**, 1151–1159 (2021).
16. Jenn, A., Azevedo, I. L. & Ferreira, P. The impact of federal incentives on the adoption of hybrid electric vehicles in the United States. *Energy Econ* **40**, 936–942 (2013).
17. Sun, X., Liu, X., Wang, Y. & Yuan, F. The effects of public subsidies on emerging industry: An agent-based model of the electric vehicle industry. *Technol Forecast Soc Change* **140**, 281–295 (2019).
18. Cottes, J. Technological variation and the US renewable fuel standard. *Technol Anal Strateg Manag* **26**, 385–399 (2014).
19. Kessler, J. & Sperling, D. Tracking U.S. biofuel innovation through patents. *Energy Policy* **98**, 97–107 (2016).
20. Lark, T. J. *et al.* Environmental outcomes of the US Renewable Fuel Standard. *Proceedings of the National Academy of Sciences* **119**, (2022).
21. Hoekman, S. K., Broch, A. & Liu, X. (Vivian). Environmental implications of higher ethanol production and use in the U.S.: A literature review. Part I – Impacts on water, soil, and air quality. *Renewable and Sustainable Energy Reviews* **81**, 3140–3158 (2018).
22. Bento, A. M., Klotz, R. & Landry, J. R. Are there Carbon Savings from US Biofuel Policies? The Critical Importance of Accounting for Leakage in Land and Fuel Markets. *The Energy Journal* **36**, (2015).
23. Hudiburg, T. W. *et al.* Impacts of a 32-billion-gallon bioenergy landscape on land and fossil fuel use in the US. *Nat Energy* **1**, 15005 (2016).
24. Rajagopal, D., Hochman, G. & Zilberman, D. Indirect fuel use change (IFUC) and the lifecycle environmental impact of biofuel policies. *Energy Policy* **39**, 228–233 (2011).
25. Gitiaux, X., Rausch, S., Paltsev, S. & Reilly, J. M. Biofuels, Climate Policy, and the European Vehicle Fleet. *Journal of Transport Economics and Policy* **46**, (2012).
26. de Gorter, H. & Just, D. R. The Economics of a Blend Mandate for Biofuels. *Am J Agric Econ* **91**, 738–750 (2009).
27. Grafton, R. Q., Kompas, T., Long, N. Van & To, H. US biofuels subsidies and CO2 emissions: An empirical test for a weak and a strong green paradox. *Energy Policy* **68**, 550–555 (2014).

28. Allaire, M. & Brown, S. P. A. The Green Paradox of U.S. Biofuel Subsidies: Impact on Greenhouse Gas Emissions. *Economics of Energy & Environmental Policy* **4**, (2015).
29. Huseynov, S. & Palma, M. A. Does California's Low Carbon Fuel Standards reduce carbon dioxide emissions? *PLoS One* **13**, e0203167 (2018).
30. Yeh, S., Witcover, J., Lade, G. E. & Sperling, D. A review of low carbon fuel policies: Principles, program status and future directions. *Energy Policy* **97**, 220–234 (2016).
31. Greene, D. L., Kahn, J. R. & Gibson, R. C. Fuel Economy Rebound Effect for U.S. Household Vehicles. *The Energy Journal* **20**, (1999).
32. Wang, J., Quiggin, J. & Wittwer, G. The rebound effect of the Australian proposed light vehicle fuel efficiency standards. *Econ Anal Policy* **61**, 73–84 (2019).
33. Yoo, S., Koh, K. W., Yoshida, Y. & Wakamori, N. Revisiting Jevons's paradox of energy rebound: Policy implications and empirical evidence in consumer-oriented financial incentives from the Japanese automobile market, 2006–2016. *Energy Policy* **133**, 110923 (2019).
34. Konishi, Y. & Zhao, M. Can Green Car Taxes Restore Efficiency? Evidence from the Japanese New Car Market. *J Assoc Environ Resour Econ* **4**, 51–87 (2017).
35. Yang, Z. & Tang, M. Welfare Analysis of Government Subsidy Programs for Fuel-Efficient Vehicles and New Energy Vehicles in China. *Environ Resour Econ (Dordr)* **74**, 911–937 (2019).
36. Du, H., Chen, Z., Zhang, Z. & Southworth, F. The rebound effect on energy efficiency improvements in China's transportation sector: A CGE analysis. *Journal of Management Science and Engineering* **5**, 249–263 (2020).
37. Ross Morrow, W., Gallagher, K. S., Collantes, G. & Lee, H. Analysis of policies to reduce oil consumption and greenhouse-gas emissions from the US transportation sector. *Energy Policy* **38**, 1305–1320 (2010).
38. Pui, K. L. & Othman, J. Economics and environmental implications of fuel efficiency improvement in Malaysia: A computable general equilibrium approach. *J Clean Prod* **156**, 459–469 (2017).
39. Yang, Z., Mock, P., German, J., Bandivadekar, A. & Lah, O. On a pathway to de-carbonization – A comparison of new passenger car CO2 emission standards and taxation measures in the G20 countries. *Transp Res D Transp Environ* **64**, (2018).
