# Supplementary material for: Technology innovation and environmental outcomes of road transportation policy instruments
Source: Nat Commun. 2025 May 14;16:4467. doi: 10.1038/s41467-025-59111-8 (PMC12078554; doi:10.1038/s41467-025-59111-8)
Supplement: Supplementary file 2 — Description of Additional Supplementary Files [file 41467_2025_59111_MOESM2_ESM.pdf]

Description of Additional Supplementary Files

File Name: Supplementary Data 1
Description: Policy Instrument Interactions

File Name: Supplementary Data 2
Description: Evidence Strength

File Name: Supplementary Data 3
Description: Coded Policy Instrument Outcomes and Impacts
