# Supplementary material for: Technology innovation and environmental outcomes of road transportation policy instruments
Source: Nat Commun. 2025 May 14;16:4467. doi: 10.1038/s41467-025-59111-8 (PMC12078554; doi:10.1038/s41467-025-59111-8)
Supplement: Supplementary file 6 — Reporting Summary [file 41467_2025_59111_MOESM6_ESM.pdf]

nature portfolio

Corresponding author(s): Clara Ma

Last updated by author(s): Feb 23, 2025

Reporting Summary

Nature Portfolio wishes to improve the reproducibility of the work that we publish. This form provides structure for consistency and transparency in reporting. For further information on Nature Portfolio policies, see our Editorial Policies and the Editorial Policy Checklist.

Statistics

For all statistical analyses, confirm that the following items are present in the figure legend, table legend, main text, or Methods section.

n/a	Confirmed	
☐	☒	The exact sample size (*n*) for each experimental group/condition, given as a discrete number and unit of measurement
☒	☐	A statement on whether measurements were taken from distinct samples or whether the same sample was measured repeatedly
☒	☐	The statistical test(s) used AND whether they are one- or two-sided<br>*Only common tests should be described solely by name; describe more complex techniques in the Methods section.*
☒	☐	A description of all covariates tested
☒	☐	A description of any assumptions or corrections, such as tests of normality and adjustment for multiple comparisons
☒	☐	A full description of the statistical parameters including central tendency (e.g. means) or other basic estimates (e.g. regression coefficient) AND variation (e.g. standard deviation) or associated estimates of uncertainty (e.g. confidence intervals)
☒	☐	For null hypothesis testing, the test statistic (e.g. *F*, *t*, *r*) with confidence intervals, effect sizes, degrees of freedom and *P* value noted<br>*Give P values as exact values whenever suitable.*
☒	☐	For Bayesian analysis, information on the choice of priors and Markov chain Monte Carlo settings
☒	☐	For hierarchical and complex designs, identification of the appropriate level for tests and full reporting of outcomes
☒	☐	Estimates of effect sizes (e.g. Cohen's *d*, Pearson's *r*), indicating how they were calculated

Our web collection on statistics for biologists contains articles on many of the points above.

Software and code

Policy information about availability of computer code

Data collection	We used Clarivate's Web of Science Core Collection to conduct our literature search for the systematic review and Mendeley Reference Manager 2.100.0 to screen and organize studies.
Data analysis	We used Microsoft Excel 16.60 to analyze evaluations of policy instruments from the systematic literature review sample.

For manuscripts utilizing custom algorithms or software that are central to the research but not yet described in published literature, software must be made available to editors and reviewers. We strongly encourage code deposition in a community repository (e.g. GitHub). See the Nature Portfolio guidelines for submitting code & software for further information.

Data

Policy information about availability of data

All manuscripts must include a data availability statement. This statement should provide the following information, where applicable:

- Accession codes, unique identifiers, or web links for publicly available datasets
- A description of any restrictions on data availability
- For clinical datasets or third party data, please ensure that the statement adheres to our policy

The datasets generated and analyzed during the current study are available as Supplementary Information.

Research involving human participants, their data, or biological material

Policy information about studies with human participants or human data. See also policy information about sex, gender (identity/presentation), and sexual orientation and race, ethnicity and racism.

Reporting on sex and gender	N/A
Reporting on race, ethnicity, or other socially relevant groupings	N/A
Population characteristics	N/A
Recruitment	N/A
Ethics oversight	N/A

Note that full information on the approval of the study protocol must also be provided in the manuscript.

Field-specific reporting

Please select the one below that is the best fit for your research. If you are not sure, read the appropriate sections before making your selection.

☐ Life sciences ☒ Behavioural & social sciences ☐ Ecological, evolutionary & environmental sciences

For a reference copy of the document with all sections, see nature.com/documents/nr-reporting-summary-flat.pdf

Behavioural & social sciences study design

All studies must disclose on these points even when the disclosure is negative.

Study description	We developed an indicator-based framework to systematically review and analyze ex ante and ex post evidence from the academic literature on the technology innovation and environmental outcomes of 14 types of road transportation policy instruments. Ex ante studies from the final systematic literature review sample typically used quantitative methods such as model simulations and stated preference surveys. Ex post studies used both qualitative and quantitative methods including case studies, panel data analysis, revealed preference surveys and consequential life cycle assessments.
Research sample	Ex ante and ex post evaluations from studies of sector-specific road transportation policy instruments on technology innovation and environmental outcomes were included, as detailed in the Methods section of our manuscript. The final systematic review sample included 468 peer-reviewed studies. This sample was chosen to allow for an analysis of the impacts of road transportation policy instruments on greenhouse gas emissions, air pollution and land use, as well as on various indicators of technology innovation.
Sampling strategy	We considered all studies that satisfied the search criteria, as defined in Supplementary Methods (SI.1).
Data collection	The data were identified, collected, screened and analyzed using the Web of Science Core Collection, Mendeley Reference Manager 2.100.0 and Microsoft Excel 16.60.
Timing	Peer-reviewed studies published between January 1, 1975 and October 31, 2022 (inclusive) were considered for inclusion.
Data exclusions	Studies which did not align with the inclusion/exclusion criteria, as defined in the Methods section, were excluded. 17,867 studies were excluded based on pre-established inclusion and exclusion criteria: First, studies where the type of policy instrument or outcome was not clearly defined, e.g. those evaluating a broad category of instruments but not specifying any concrete type of policy instrument, were excluded from the final sample. Second, studies where the policy instruments making up a policy mix could not be distinguished were excluded, i.e., studies evaluating policy portfolios or packages that do not identify specific policy instruments. Third, studies focusing on the environmental outcomes of a technology without reference to a specific policy instrument were excluded. Fourth, studies where the only benchmark of comparison is another policy instrument were excluded. Lastly, voluntary agreements, local-level policy instruments focusing on traffic and congestion, e.g. low-emissions zones, and general economy-wide policies, for example, carbon pricing covering multiple sectors, were excluded.
Non-participation	Non-participation was not applicable to our study as no participants were involved in the study.
Randomization	Randomization was not applicable in the context of our study.

Reporting for specific materials, systems and methods

We require information from authors about some types of materials, experimental systems and methods used in many studies. Here, indicate whether each material, system or method listed is relevant to your study. If you are not sure if a list item applies to your research, read the appropriate section before selecting a response.

Materials & experimental systems

n/a	Involved in the study
☒	☐ Antibodies
☒	☐ Eukaryotic cell lines
☒	☐ Palaeontology and archaeology
☒	☐ Animals and other organisms
☒	☐ Clinical data
☒	☐ Dual use research of concern
☒	☐ Plants

Methods

n/a	Involved in the study
☒	☐ ChIP-seq
☒	☐ Flow cytometry
☒	☐ MRI-based neuroimaging

Plants

Seed stocks	N/A
Novel plant genotypes	N/A
Authentication	N/A
